# Heme rescues a two-component system *Leptospira biflexa *mutant

**DOI:** 10.1186/1471-2180-8-25

**Published:** 2008-01-30

**Authors:** Hélène Louvel, Jean-Michel Betton, Mathieu Picardeau

**Affiliations:** 1 Unité de Biologie des Spirochètes, Institut Pasteur, Paris, France; 2 Unité de Biochimie Structurale & CNRS URA2185, Institut Pasteur, Paris, France

## Abstract

**Background:**

Heme is typically a major iron source for bacteria, but little is known about how bacteria of the *Leptospira *genus, composed of both saprophytic and pathogenic species, access heme.

**Results:**

In this study, we analysed a two-component system of the saprophyte *Leptospira biflexa*. *In vitro *phosphorylation and site-directed mutagenesis assays showed that Hklep is a histidine kinase which, after autophosphorylation of a conserved histidine, transfers the phosphate to an essential aspartate of the response regulator Rrlep. Hklep/Rrlep two-component system mutants were generated in *L. biflexa*. The mutants could only grow in medium supplemented with hemin or δ-aminolevulinic acid (ALA). In the pathogen *L. interrogans*, the *hklep *and *rrlep *orthologous genes are located between *hemE *and *hemL *genes, which encode proteins involved in heme biosynthesis. The *L. biflexa hklep *mutant could be complemented with a replicative plasmid harbouring the *L. interrogans *orthologous gene, suggesting that these two-component systems are functionally similar. By real-time quantitative reverse transcription-PCR, we also observed that this two-component system might influence the expression of heme biosynthetic genes.

**Conclusion:**

These findings demonstrate that the Hklep/Rrlep regulatory system is critical for the *in vitro *growth of *L. biflexa*, and suggest that this two-component system is involved in a complex mechanism that regulates the heme biosynthetic pathway.

## Background

Leptospires are spirochetes divided between saprophytic and pathogenic species that remain poorly understood because of the limited availability of genetic tools. For example, targeted gene inactivation is not feasible in pathogenic *Leptospira *species.

Iron is an essential nutrient for the growth of leptospires like for most of the bacteria and the ability to acquire iron certainly contributes to the virulence of pathogenic bacteria. We previously showed that leptospires can take up a wide diversity of iron sources including heme, which is the most abundant iron source in host [1]. We also showed that *L. biflexa *possesses genes encoding putative heme acquisition systems such as TonB-dependent receptors and ABC transporters and a putative heme oxygenase that could allow the release of iron from heme [2]. Heme is a ubiquitous molecule involved in many major cellular processes. For example, heme is a cofactor for catalases and peroxidases and is an integral component of the electron transport chain where it serves as an electron carrier for cytochromes. *Leptospira *spp. possess genes that encode enzymes for the heme biosynthetic pathway unlike the spirochetes *Borrelia burgdorferi *and *Treponema pallidum*. In a previous study [3], we showed that inactivation of *hemH *in *L. biflexa*, which encodes the ferrochelatase that introduces one iron molecule into the porphyrin ring, generates heme requirement. Taken together, our previous results argue that hemin is an iron source as well as an heme source for leptospires. Free heme is toxic to bacterial cells, so heme biosynthesis is usually tightly regulated. However, little is known about how organisms regulate both the acquisition of exogenous heme and synthesis of heme [4].

We used transposon mutagenesis to generate a mutant library in the saprophyte *L. biflexa *that we screened onto media with or without hemin supplementation [2]. A heme-requiring mutant was isolated with an insertion in a gene that was predicted to encode the regulator of a two-component system. Such systems regulate gene expression in many bacteria in order to support a physiological response. Two-component systems detect a wide variety of environmental signals, including iron availability [5,6]. These systems typically comprise a membrane-associated sensor kinase and a cytoplasmic transcription regulator. The sensor autophosphorylates in response to stimuli and then transfers the phosphate group to the response regulator, which binds to specific promoters and, thus, acts as a transcriptional regulator [7]. Two-component systems are involved in many processes such as motility, virulence gene expression, and oxidative stress response [7].

In this study, we investigated the biochemical and physiological properties of this two-component system of *L. biflexa *to elucidate its role in heme metabolism.

## Results

### *L. biflexa hklep *and rrlep mutants only grow with heme and heme precursor

**Figure 1:**
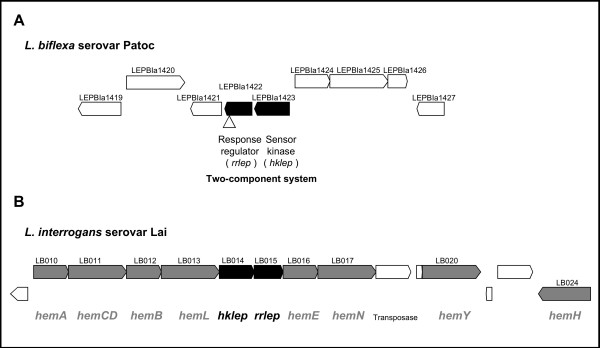
**Genetic organization of the Hklep/Rrlep two-component system in *Leptospira spp***. (A) Organization of *hklep*/*rrlep *genes in *L. biflexa*. The two-component system genes are shaded in black. Gene labels are given for each surrounding genes (white). The arrow indicates the insertion site of the *Himar1 *transposon. (B) Schematic representation of the heme biosynthesis genes in *L. interrogans*. The *hemACDBLENYH *genes are shaded in grey. The homologous two-component system genes, *hklep *and *rrlep*, are shaded in black. The *L. biflexa *Hklep and Rrlep proteins share 66 % and 69 % similarities with *L. interrogans *LB014 and LB015, respectively.

**Figure 2:**
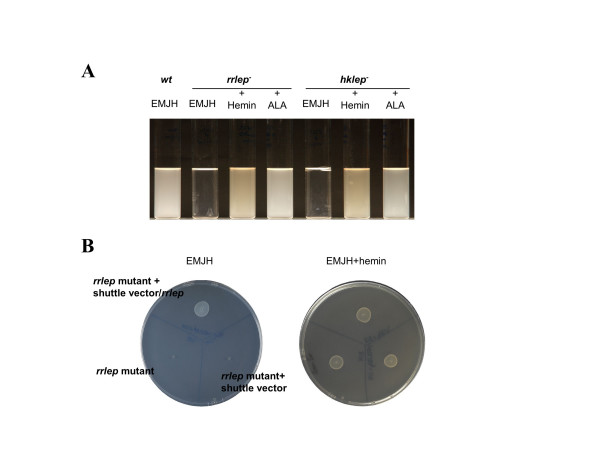
**Growth of *L. biflexa hklep *and *rrlep *mutant strains in EMJH medium**. A. The *rrlep *and *hklep *mutants grew as well as the wild-type strain (*wt*) in EMJH liquid medium supplemented with hemin or δ-aminolevulinic acid (ALA). However, the mutants did not grow in EMJH liquid medium. B. Complementation of the *L. biflexa rrlep *mutant. Transformation of *L. biflexa rrlep *mutant with the empty shuttle vector and the shuttle vector carrying the *L. biflexa rrlep *gene plated onto EMJH and EMJH supplemented with hemin. The growth of the *rrlep *mutant complemented by the *rrlep *wild type gene was similar to the wild type growth onto EMJH plates. Strains were incubated at 30°C for one week.

*Himar1 *mutagenesis was previously used to identify genes involved in heme utilization by *L. biflexa *[2]. One of the transposon mutants, which was only able to grow in EMJH supplemented with hemin, but not in EMJH alone, exhibited the transposon into a putative gene encoding the response regulator of a two-component system (Figure 1A). RT-PCR results showed that the two genes, *rrlep *and *hklep*, of this putative two-component system are co-transcribed as an operon in *L. biflexa *(data not shown). To investigate the functions of these two-component signalling genes in *L. biflexa*, we constructed *rrlep *and *hklep *deletion mutants by targeted gene replacement. Again, the resulting mutant strains were unable to grow on EMJH medium unless exogenous hemin was added (Figure 2). These mutants were also able to grow in EMJH medium supplemented with haemoglobin and δ-aminolevulinic acid (ALA) a key intermediate in heme biosynthesis pathway, but not with other potential sources of iron, e.g. iron salts or desferrioxamine. These results then indicate that only a source of heme or a precursor could support the growth of the mutant strains in EMJH (Figure 2).

**Figure 3:**
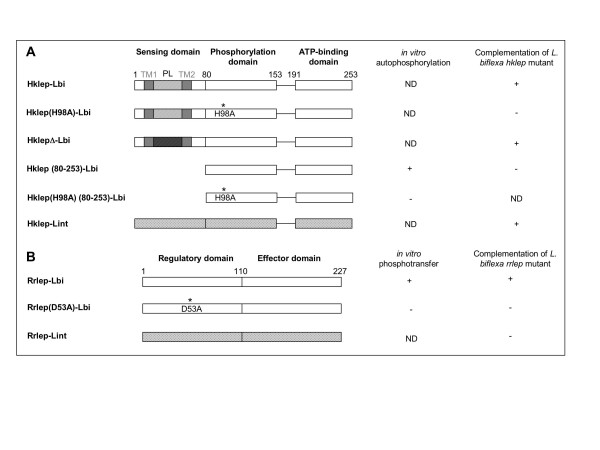
**Schematic representation of Hklep and Rrlep proteins tested for phosphorylation assays and complementation of *L. biflexa *mutants**. (A) The *L. biflexa *Hklep protein is composed of three domains. Hklep is putatively anchored into the inner membrane through two transmembrane segments (TM1-TM2) separated by a periplasmic loop (PL) that could correspond to the sensing domain. The residue histidine 98 is predicted to be the site of phosphorylation. The asterisk marks its mutation into an alanine residue in Hklep(H98A). In HklepΔ, the periplasmic loop is replaced by another sequence (see Materials and Methods). In Hklep (80–253), the putative sensing domain is deleted and the original promoter of *hklep *is replaced by a leptospiral promoter. (B) The *L. biflexa *Rrlep protein is composed of two domains. The residue aspartate 53 is predicted to be the site of phosphorylation. The asterisk marks its mutation into an alanine residue in Rrlep(D53A). Lbi and Lint refer to the *L. biflexa *and *L. interrogans *alleles, respectively. "-" indicates no *in vitro *phosphorylation or absence of complementation of the *L. biflexa *mutant strain. "+" indicates *in vitro *phosphorylation or complementation of the *L. biflexa *mutant strain. ND: not determined. The expression of HklepH98A, Hklep(80–253), Rrlep(D53A), or Rrlep-Lint (LB015) failed to complement their respective mutant and was characterized by an absence of growth (-) in EMJH medium like observed for *hklep *and *rrlep *mutants. The expression of HklepΔ or Hklep-Lint (LB014) complemented *hklep *mutant restoring a wild type growth (+) in EMJH medium.

Transformation of each mutant strain with the replicative plasmid harbouring the respective two-component signalling gene of *L. biflexa *restored a wild-type growth in EMJH medium (Figures 2 and 3). These results indicate that the growth defect was due to the inactivation of the two-component system and not to second-site mutations. It also suggests that there is no cross-talk between regulators and kinases of other cognate pairs of two-component systems in the bacterium. We concluded that the system has a crucial role in the survival of leptospires in EMJH and for heme supplying.

### The homologous two-component system of the pathogen *L. interrogans *is genetically linked to heme biosynthesis genes

The complete genome sequences of four pathogenic *Leptospira *are available, namely *L. interrogans *serovar Lai, *L. interrogans *serovar Copenhageni, and two strains of *L. borgpetersenii *serovar Hardojobovis [8-10]. The *L. biflexa *two-component system was found in these four pathogenic strains and the proteins share 66% and 69% similarities with LB014 sensor and LB015 regulator of *L. interrogans*, respectively (Figure 1B). Surprisingly, the two-component system found in the pathogens is genetically linked to the genes of the heme biosynthesis pathway, as it is also the case in the phylogenetically distant deltaproteobacteria *Bdellovibrio bacteriovorus *(data not shown). These genomic data may support a functional association between the two-component system and the biosynthesis of heme. The *L. biflexa hklep *mutant was complemented by the putative sensor gene of *L. interrogans *(LB014) carried on the shuttle vector (Figure 3). However, expression of the regulator gene of *L. interrogans *(LB015) of both LB014 and LB015 (data not shown) in the *L. biflexa rrlep *mutant did not restore wild-type growth (Figure 3). This may indicate that the heterologous expression of the *L. interrogans *regulator does not allow interactions with the *L. biflexa *sensor or *L. biflexa *target DNAs. In conclusion, the orthologous two-component systems in *L. biflexa *and *L. interrogans *are partially replaceable and functionally similar.

### Signal transduction through Hklep/Rrlep relies on phosphorylation capabilities

**Figure 4:**
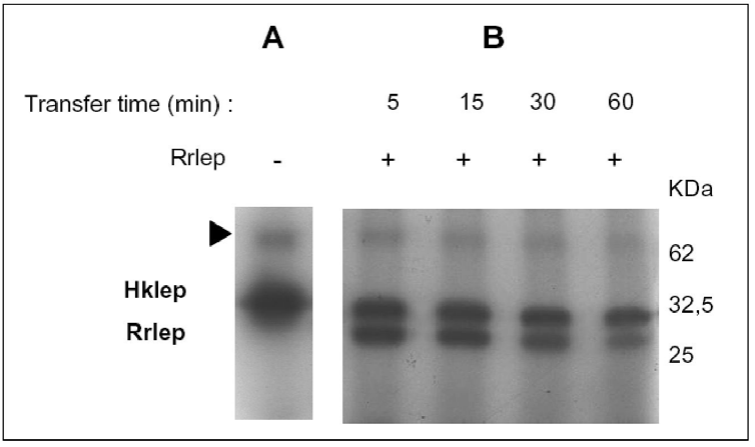
**Phosphorylation assays with *L. biflexa *Hklep and Rrlep proteins**. (A) For autophosphorylation assays, Hklep was incubated with [γ-^32^P]ATP for 1 h 30. The arrow indicates the putative dimer form of Hklep. (B) For phosphotranfer assays, an equal amount of Rrlep protein was added to the phosphorylated Hklep and the reaction was further incubated for 5, 15, 30, and 60 min. Proteins were separated on 12 % SDS-polyacrylamide gel and visualized using autoradiography. The molecular mass is indicated in kDa.

**Figure 5:**
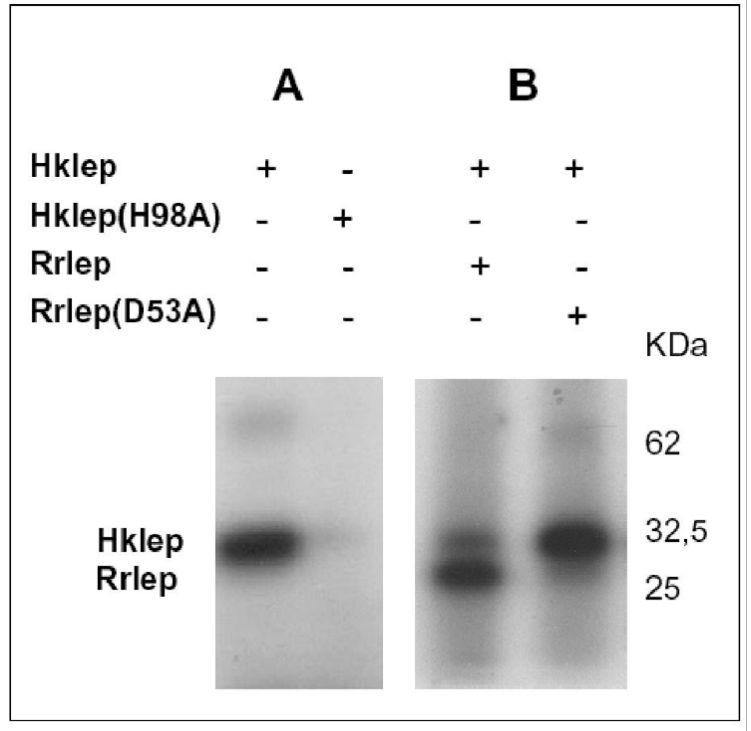
**Phosphorylation assays of *L. biflexa *Hklep and Rrlep mutant proteins**. (A) Site-directed mutagenesis of the *L. biflexa *histidine kinase protein. The Hklep or Hklep(H98A) proteins were incubated with [γ-^32^P]ATP for 1 h 30. (B) Site-directed mutagenesis of the *L. biflexa *response regulator protein. Hklep was incubated with [γ-^32^P]ATP for 1 h 30 and an equal amount of Rrlep or Rrlep(D53A) proteins was added. The reaction was further incubated for 1 h. Proteins were separated on 12 % SDS-polyacrylamide gel and visualized using autoradiography. The molecular mass is indicated in kDa.

Sequence analysis of the two-component system of both *L. biflexa *and *L. interrogans *reveals that it is a classical His/Asp-dependent two-component system consisting of a histidine protein kinase and a response regulator with receiver and transmitter domains. The first reaction in the signalling cascade consists in the autophosphorylation of a highly conserved histidine residue of the histidine kinase. By incubating with [γ-^32^P]ATP, the recombinant Hklep protein was rapidly phosphorylated (Figure 4a). The phosphorylation assays also allowed the observation of a secondary signal corresponding to twice the expected size of Hklep, suggesting that Hklep may form homodimers (Figures 4 and 5). Following the autophosphorylation of the sensor kinase, the phosphate group is typically transferred to an aspartyl residue of the conserved N-terminal receiver domain of the response regulator. By incubating Rrlep with the phosphorylated Hklep, we observed both phosphorylation of the regulator and reduction of the phosphosignal of the kinase (Figure 4B). The amount of labelled Rrlep also decreased during the course of the experiment, possibly due to unstable phosphorylation.

Based on sequence alignments of other two-component systems, the histidine residue at position 98 in Hklep was predicted to be the auphosphorylation site, and the aspartate D53 of Rrlep the site of phosphotransfer (data not shown). To confirm the identity of the phosphate-donor and -receiver residues, we mutated the H98 and D53 of Hklep and Rrlep, respectively, by site-directed mutagenesis. *In vitro *phosphorylation assays showed that Hklep(H98A) was not able to autophosphorylate anymore (Figures 3 and 5) and that Rrlep(D53A) was not subject to phosphotransfer and did not stimulate the dephosphorylation of the kinase (Figures 3 and 5). In addition, the mutated alleles were unable to complement the *hklep *and *rrlep *mutant strains (Figure 3). In conclusion, H98 and D53 are key residues for the phosphorylation mechanisms of the *L. biflexa *Hklep/Rrlep two-component system.

### Rrlep/Hklep may regulate the expression of heme biosynthesis genes

The C-terminal region of Rrlep (residues 110 to 227) is predicted to contain a helix-turn-helix DNA-binding motif. The response regulator may bind to target DNAs via this domain while being phosphorylated. Furthermore, two-component response regulators typically autoregulate their own expression. Quantitative Reverse Transcription-PCR (qRT-PCR) assays showed that inactivation of *rrlep *gene in *L. biflexa *led to a four-fold decrease in transcript level of *hklep *(data not shown). The expression of this two-component system is therefore likely to be autoregulated by the response regulator. No significant change was observed in the relative expression of the two-component signalling genes in the wild-type strain cultivated with or without hemin or δ-aminolevulinic acid (data not shown). Taken together, these results suggest that another regulator is involved in the regulation of this two-component system.

**Figure 6:**
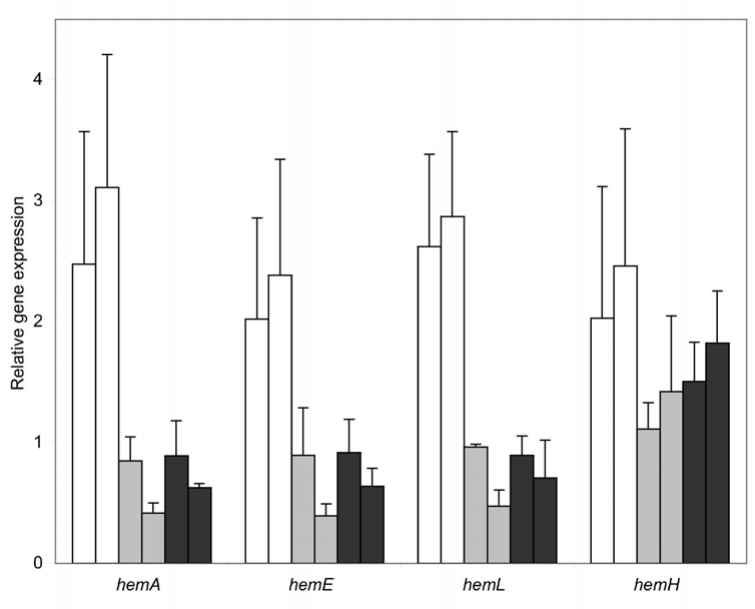
**Relative gene expression of *L. biflexa hemA*, *hemE*, *hemL*, and *hemH *genes measured by real-time quantitative RT-PCR**. The levels of specific mRNA transcript of *hemAELH *genes were quantified in wild-type (bars in white), *rrlep *mutant (bars in grey), and *hklep *mutant (bars in black) grown in EMJH supplemented with 2 (left bar) or 10 μM (right bar) hemin. The amounts of mRNA transcript are shown relative to the quantity of that particular mRNA transcript in EMJH condition (without hemin). As an endogenous control, the 23S rRNA (23S rRNA = 1.0) was used for normalization of transcript levels. Experiments were performed in triplicate from distinct cultures to establish standard deviations.

Since the *L. interrogans *two-component system (LB014-LB015) is clustered with heme biosynthetic genes (Figure 1), we investigated the expression of *L. biflexa hem *genes in response to heme availability by qRT-PCR. We compared the expression of *hemL*, leading to δ-aminolevulinic acid synthesis, *hemE*, *hemA *and *hemH *in *L. biflexa *wild-type and mutant strains in media supplemented with low (2 μM) or high (10 μM) level of hemin (Figure 6). Our results showed that *hemAELH *expression was 2 to 4 fold higher with hemin than without hemin in the wild-type strain of *L. biflexa *(data not shown). Expression of *hemAEL *was significantly decreased, from 2 to 10 fold, in *hklep *and *rrlep *mutants relative to the wild-type strain. Conversely, *hklep *and *rrlep *mutations displayed weaker effects on *hemH *expression. We concluded that heme biosynthesis genes may be regulated in response to heme availability. The Hklep/Rrlep two-component system may regulate *hemAEL *genes but not, or differently, *hemH*, which is transcribed in the opposite direction and then apart from the heme biosynthetic genes.

### The periplasmic loop of the kinase is not essential but the cytoplasmic region is not sufficient to ensure physiological function

Hklep is a sensor histidine kinase with two predicted N-terminally located transmembrane segments that may anchor the protein to the cytoplasmic membrane of *L. biflexa*. The extracytoplasmic region between transmembrane segments is usually involved in the detection of environmental stimuli in other sensor kinases. Since we assumed that the periplasmic loop region of Hklep is involved in the detection of the environmental signal, the corresponding 32 amino acids (residues 31 to 62) were replaced by site-directed mutagenesis. The resulting plasmid construct was then used to complement the *L. biflexa hklep *mutant. The *hklep *gene lacking the original periplasmic loop was still able to complement the *hklep *mutant, i.e. it restores a wild-type growth in EMJH without hemin (Figure 3). This suggests that the periplasmic loop is not involved in the recognition of the signal. Nevertheless, *L. biflexa hklep *mutant could not be complemented with a Hklep protein lacking both the predicted transmembrane segments and the periplasmic loop (Figure 3).

## Discussion

To isolate hemin-requiring mutants, we used transposon mutagenesis in *L. biflexa *and identified the Hklep/Rrlep two-component system. In this study, we showed that Hklep is a histidine kinase which, after autophosphorylation of H98, transfers the phosphate to D53 of the response regulator Rrlep.

The mutants of this two-component system can only grow in medium supplemented with hemin or δ-aminolevulinic acid (ALA) but not with other iron sources, suggesting that the mutants need to acquire exogenous hemin as an heme source instead of an iron source. This also suggests that Hklep/Rrlep contributes to the regulation of steps leading to ALA synthesis. Although significant differences were detected in the relative expression of the *hemAEL *genes in the mutant versus the wild-type, we do not have conclusive evidence for direct regulation of *hem *genes by the two-component system. We postulate that the two-component response regulator may be connected in interacting networks and cascades of regulation.

Most bacteria possess many two-component systems, and the *L. interrogans *genome is predicted to encode 25 [11], that are required to control the activity of many genes in response to a variety of environmental signals. A key aspect of any regulatory system is the identity of the environmental signal to which the system responds. Our data suggest that the Hklep/Rrlep system senses environmental and/or intracellular heme. Two-component system kinases are supposed to detect environmental signals through their periplasmic domain [7]. Our results showed that the predicted periplasmic loop is not essential for the function purpose of the system. However, it remains possible that the N-terminal domain of Hklep, including the putative membrane-spanning regions, plays a role in signal detection.

Biosynthesis of porphyrins and related compounds proceeds via a common set of intermediates from ALA through the first cyclic tetrapyrrole, uroporphyrinogen III, at which point the pathway splits into two branches, one leading to reduced products such as siroheme and vitamin B12 and the other leading to oxidized end products, including hemes, bilins, and bacteriochlorophylls. The expression of *hem *genes is usually transcriptionally regulated in response to exogenous heme concentration [4]. In *L. biflexa*, the transcription of the *hem *genes that we studied is significantly increased on hemin addition. Hklep/Rrlep may be, at least indirectly, responsible for the regulation of several *hem *genes.

The orthologous two-component systems in pathogenic *Leptospira *spp. are arranged in a novel fashion, distinct from that in typical bacterial heme biosynthetic loci, and this provides genetic evidence for *hem *reorganization in *Leptospira*. Transcription analyses in *L. interrogans *showed that *hemE *and *hemL *are co-transcribed with *hklep *and *rrlep*, consistent with an operon organization of *hem *and two-component signalling genes (data not shown). The biological significance of this gene association remains open, but strengthens the role of the two-component system in heme metabolism.

## Conclusion

The *in vivo *characterization of *L. biflexa hklep *and *rrlep *mutants argues for a role for this regulatory system in heme metabolism. However, the precise role of this two-component system remains unclear and further investigations will be necessary for identifying both its signal and target. Further investigation should also include the study of biochemical properties and transcriptional regulation of the homologous two-component system in the pathogens. As heme transport, storage, and metabolism are important for pathogenic bacteria [12], this system might contribute to the pathogenicity potential of leptospires.

## Methods

### Bacterial strains and growth conditions

*Leptospira biflexa *serovar Patoc strain Patoc I and *L. interrogans *serovar Lai strain Lai were grown at 30°C in EMJH [13,14]. When necessary, media were supplemented with 2 to 10 μM hemin, 10 μM haemoglobin, or 50 μM δ-aminolevulinic acid (ALA). Kanamycin and spectinomycin were added at 40 μg ml^-1^. *Escherichia coli *XL10 (Invitrogen) and *E. coli *RosettaBlue(DE3) (Novagen) were grown at 37°C in Luria-Bertani (LB) medium supplemented with the appropriate antibiotics and chemicals.

### DNA/RNA manipulations

Genomic DNA of leptospires was isolated as previously described [15]. Plasmid DNA was purified using the Qiagen Plasmid Miniprep Kit (Qiagen GmbH, Hilden, Germany).

For transcription studies, RNAs were isolated from exponential phase cultures of *L. biflexa *grown in EMJH, EMJH with 2 or 10 μM hemin or EMJH after an overnight incubation of the cells with 0.25 mM 2,2'-dipyridyl. Total RNAs were extracted using Tri-reagent solution and DNase I treatment following the manufacturer recommendations (Ambion Inc.). Absence of DNA contamination was checked by PCR. RNA concentrations were measured by spectrophotometry at 260 nm. RNAs were analyzed by RT-PCR using SuperScript One Step RT-PCR with Platinium Taq (Invitrogen). Quantitative reverse-transcription PCR was performed in two steps as previously described [16]. Data from amplifications were analysed with the quantification program RelQuant (Roche). The relative expression of the target genes was normalized to the 23S rRNA. Quantitative expressions were evaluated in triplicate from three independent RNA extractions.

### Recombinant protein expression and purification

To overproduce the proteins in *E. coli*, the full-length *rrlep *and the truncated *hklep *(region corresponding to residues 90–300) coding sequences from *L. biflexa *were amplified by PCR (nucleotide sequences of primer pairs available on request) and cloned into the *Hind*III and *Xho*I restriction sites of pET30a(+) (Novagen), generating pETrrlep and pEThklep, respectively. *E. coli *RosettaBlue(DE3) strain (Novagen) harbouring the pET30a(+)-based constructs were grown in LB supplemented with 25 μg chloramphenicol ml^-1 ^and 50 μg kanamycin ml^-1 ^at 37°C to a OD_600 _of 0.6–0.8. IPTG (isopropyl-β-D-thiogalactopyranoside) was added at a final concentration of 1 mM and the cultures were further incubated for several hours. *E. coli *cells were washed into lysis buffer (50 mM NaH_2_PO_4_, 300 mM NaCl, 10 mM imidazole), lyzed by freezing and thawing followed by sonication, then centrifuged at 10,000 × g for 20 min at 4°C. The supernatant was recovered and incubated with Ni^2+^-agarose beads (Qiagen). In every case, the purity of the studied proteins was > 90 %, as judged by Coomassie-stained SDS-PAGE gels. Protein concentration was determined by Bradford protein assay (Biorad). To differentiate the proteins on gel, we produced Hklep with a N-terminal His-tag (31 kDa) and Rrlep without a His-tag (27 kDa). His-tagged proteins were eluted using a 250 mM imidazole-containing buffer. His-tag was removed by incubation with enterokinase as recommended by the manufacturer (Novagen).

### Mutagenesis of the Hklep and Rrlep proteins

The *hklep *and *rrlep *genes of *L. biflexa *were cloned into the spectinomycin-resistance conferring *L. biflexa*-*E. coli *shuttle vector pGSLEP [17] to generate pGSLhklep and pGSLrrlep, respectively. Site-directed mutagenesis of Hklep (LEBBIa1423) and Rrlep (LEPBIa1422) was performed by PCR amplification of pGSLhklep/pGSLrrlep or pEThklep/pETrrlep vectors using oligonucleotide primer pairs carrying the mutations and *Pwo *polymerase (Roche). The chosen mutations consisted in replacing the histidine kinase H98 and the response regulator D53 by an alanine leading to either pGSLhklep(H98A) and pEThklep(H98A) or pGSLrrlep(D53A) and pETrrlep(D53A), respectively. The periplasmic loop of the kinase protein (pGSLhklepΔ) was replaced by using a two-steps overlap method. Region of Hklep comprised from the amino acids F31 to S62 was replaced by the sequence N-GSA SAG ASG SGA-C (encoded by the underlined sequences of the primers, see below). Nucleotides from 1 to 93 and from 186 to stop codon were amplified separately with the following primer pairs 5'-TGCAAACGAAGCTCCAGCGGAG-3'/5'-tgctccagatccagatgctcctgcagatgcagatccCCCTAAAATCCACCACCAGAC-3' and 5'-ggatctgcatctgcaggagcatctggatctggagcaTTTTTCCTTTCGATGTTAACAC-3'/5'-TGTTTTGGCCCACTCTACACGG-3', respectively, and *Pfx *polymerase (Invitrogen). PCR products were then used as templates to generate the full-length coding sequence. Hklep protein was also restricted to its cytoplasmic domain (Hklepc) and the truncated *hklep *gene (residues 90 to 300) was fused to *L. interrogans hsp10 *promoter (pGSLPhklepc). The expression and *in vivo *stability of Hklepc were confirmed by western blotting using anti-His tag antibodies (data not shown).

### Gene inactivation and complementation of *L. biflexa *strains

A 6,000 mutant library of *L. biflexa *was generated by random transposon mutagenesis using *Himar1 *as previously described [2]. The mutants were screened onto media with and without 10 μM hemin and we identified the mutants that were not able to grow without hemin. The transposon insertion site was identified by LM-PCR as previously described [18]. Targeted gene inactivation in *L. biflexa *was carried out as previously described [15]. The recombinant vectors pGSLrrlep, pGSLhklep, pGSLrrlep(D53A), pGSLhklep(H98A), pGSLhklepΔ, and pGSLPhklepc were used for functional complementation of the *L. biflexa *mutant strains. *L. interrogans *serovar Lai homologous genes (LB014 and LB015) were also cloned into pGSLEP [17] and tested for complementation. All plasmid constructs were checked by sequencing.

### In vitro phosphorylation assays

Proteins were dialyzed against the phosphorylation buffer (50 mM Tris-HCl, pH 7.5, 5 mM MgCl_2_, 50 mM KCl, 1 mM DTT, and 10 % glycerol). For autophosphorylation assays, 4–6 μM of Hklep or Hklep(H98A) were incubated with 0.4 μM ATP containing 20 μCi [γ-^32^P]ATP for 1 h 30 at room temperature. For phosphotransfer assays, 4–6 μM of Rrlep or Rrlep(D53A) were added to Hklep. The reaction was further incubated for 5 min to 1 h and stopped by SDS buffer. Proteins were then resolved on 12 % SDS-polyacrylamide gel electrophoresis and visualized using Coomassie blue staining or X-ray autoradiography.

### Sequence analysis and nucleotide sequence accession number

The DNA sequence data were analyzed with BLAST [19], SMART [20], and TMPRED [21] programs. The nucleotide sequence of the locus containing the *L. biflexa *two-component signalling genes is deposited under accession number EF577043.

## Authors' contributions

HL, JMB, and MP conceived the study and experimental design and drafted the manuscript. HL carried out the experiments. All authors read and approved the final manuscript.
